# Child marriage among boys in high-prevalence countries: an analysis of sexual and reproductive health outcomes

**DOI:** 10.1186/s12914-019-0212-8

**Published:** 2019-08-16

**Authors:** Christina Misunas, Colleen Murray Gastón, Claudia Cappa

**Affiliations:** Data and Analytics Section, Division of Data, Research and Policy, UNICEF, New York, USA

**Keywords:** Child marriage, Adolescent boys, Male sexual and reproductive health

## Abstract

**Background:**

While the determinants and impacts of child marriage among girls have been well documented, little research exists on the practice among boys. This paper explores the sociodemographic profile of men who married by age 18 and assesses whether they are more or less advantaged than their peers in terms of their sexual and reproductive health outcomes.

**Methods:**

This analysis used the most recent data from nationally representative household surveys for the 15 countries with the highest prevalence of marriage by age 18 among men aged 20–24 at the time of the survey. The prevalence of child marriage was then explored for the full cohort of men aged 20–49 through descriptive statistics and bivariate analysis. Available reproductive health indicators were explored, comparing men who married during childhood and men who married in adulthood. For the youngest and oldest cohorts, the total number of children fathered and the total ideal number of children were compared based on whether men married by age 18.

**Results:**

For this subset of countries, the prevalence of child marriage among men aged 20–24 ranges from 8.4 to 27.9%. The practice appears most common among respondents living in the poorest households and in rural areas, and with no education or only primary schooling. Men who married as children appear less likely to have comprehensive knowledge of HIV than their peers who married in adulthood. Little difference among men who married by age 18 and those who married in adulthood was observed regarding knowledge or use of modern methods of contraception. In almost all countries with data, the odds of having fathered three or more children among men aged 20–29 are higher for those who married as children compared to their peers. In four countries, the odds of exceeding one’s ideal family size among men aged 40–49 also appear higher among those who married during childhood compared to men who married at older ages.

**Conclusion:**

These results highlight the need for further research to identify drivers of the practice and short- and long-term outcomes for men who married during childhood, specifically concerning fatherhood, fertility preferences, and completed family size.

## Introduction

### Background

Child marriage, defined as a formal marriage or informal union before the exact age 18, has been widely acknowledged as a violation of fundamental human rights by several conventions, treaties, and international agreements, including the Convention on the Rights of the Child, the Convention on the Elimination of All forms of Discrimination against Women, and the Universal Declaration of Human Rights [1–4].

Yet the mere existence of legal instruments prohibiting child marriage adopted by the international community has not been enough to eliminate the practice. Although age 18 is recognized as the *age of the majority* in most parts of the world, the Convention on the Rights of the Child creates exceptions for national laws [3]. Moreover, even in some countries with strong legal frameworks prohibiting the practice, implementation of the ban on child marriage is often inconsistent and weak [5]. Despite a general global trend towards later marriage for both sexes [6], the percentage of girls and boys who marry during childhood remains sizeable. Globally, an estimated 21.2% of females currently aged 20–24 were first married or in union before age 18; 4.5% of males currently aged 20–24 were also married during childhood based on data available for 82 countries [7].

Research on the practice of child marriage has focused mainly on its determinants and outcomes for girls and the children they bear [8–12]. In many countries, the risk of child marriage is highest for girls in rural areas, in the poorest communities, and with lower levels of education [8, 13]. Likewise, research on health outcomes of the practice has indicated that girls who marry during childhood are often at higher risk of unintended pregnancy, acquiring HIV and other sexually transmitted infections, pregnancy-related mortality and morbidity, and potential longer-term consequences of early childbearing such as obstetric fistula and cervical cancer [14–16]. In comparison to marriage that occurs during adulthood, child marriage has been shown to be associated with lower age at first birth, lower contraceptive use, higher fertility, and higher risk of having more than one’s ideal number of children [17, 18].

Conversely, less is known about the profile of men who married during childhood and the determinants of the practice among boys; no comparable research currently exists on the long-term reproductive health and fertility outcomes for men who married before turning 18. The lack of research is likely due to differences in the global magnitude of the practice [6–8] and the grave physiological risks of early pregnancy and childbirth often faced by girls [18, 19]. However, the practice remains a rights violation for children of both sexes and further investigation on the scale and implications of child marriage among boys is needed.

Similar to girls who marry during childhood, boys who marry before age 18 might enter into unions that involve experiences and responsibilities, including early fatherhood and providing for the household, for which they may lack adequate knowledge, resources, and psychosocial support. Both in the immediate aftermath and later in life, men who married as children might suffer similar reproductive health consequences regarding lower knowledge and use of contraception and higher unwanted fertility as women married during childhood. While men’s contraceptive needs and fertility intentions have been explored more broadly [20–24], to the best of our knowledge, no studies have assessed whether and how they vary based on age at marriage.

### Aims and objectives

This paper aims to explore the sociodemographic background of men who married during childhood in countries where the practice is most common to determine if prevalence is concentrated in certain subpopulations and whether the factors strongly associated with child marriage among girls, such as residence, wealth, and education, are similar for boys. Subsequent analysis provides insight into whether key life outcomes related to sexual and reproductive health and family size differ for men who married as children compared to their peers who married in adulthood.

## Methods

### Data sources

Data used for this analysis were from nationally representative household surveys, predominantly the Multiple Indicator Cluster Surveys (MICS), supported by the United Nations Children’s Fund (UNICEF), and the Demographic and Health Surveys (DHS), supported by the United States Agency for International Development (USAID), both of which use two-stage cluster sampling.

Nationally representative data collected during the past decade on child marriage among boys were available for 82 countries. To identify countries where the practice among boys is most common, the prevalence of marriage by age 18 among men currently aged 20–24 was used as a proxy for the current estimate because this cohort most recently completed exposure to the risk period. Based on the latest estimates, the following 15 countries with the highest prevalence of child marriage among men currently aged 20–24 were selected for further analysis: the Plurinational State of Bolivia (DHS 2008), the Central African Republic (MICS 2010), Comoros (DHS 2012), Cuba (MICS 2014), Guatemala (DHS 2015), Guyana (MICS 2014), Honduras (DHS 2011–2012), Lao People’s Democratic Republic (MICS 2011–2012), Madagascar (Enquête Nationale sur le Suivi des Objectifs du Millénaire pour le Développement à Madagascar (2013)), the Marshall Islands (DHS 2007), Mozambique (DHS 2011), Nauru (DHS 2007), Nepal (DHS 2016), Nicaragua (Encuesta Nicaragüense de Demografía y Salud (2011–2012)), and Thailand (MICS 2015). The Marshall Islands and Nauru were excluded from further analysis because the data are not publicly available and request for access was not granted.

In these surveys, males aged 15–49 in selected households were identified as respondents to individual questionnaires. Among other topics, men consenting to be interviewed were asked whether they had ever been married or lived with someone as if married. Men who responded affirmatively were asked to provide the month and year they began living with their first partners and, for validation, their exact age at first marriage or union [25, 26].

Although the prevalence of child marriage among men currently aged 20–24 was used to identify countries for further investigation, subsequent analyses presented in this paper included the full cohort of men aged 20–49, 20–29 or 40–49 to ensure the analytic sample was of sufficient size. The 15–19 age group was excluded due to censoring.

### Outcome-level indicators

Prevalence of marriage by age 18 among men aged 20–49 was explored using descriptive statistics and bivariate analysis of age and other background-level indicators for which research on child marriage among girls has revealed associations. These variables include household wealth (quintiles), place of residence (urban and rural), and level of education attainment (none or primary and secondary or higher). Prevalence estimates for each five-year age cohort from the most recent available data source were also compared to assess trends.

Two available indicators of reproductive health (comprehensive knowledge of HIV,^1^ and knowledge and use of modern methods of contraception^2^) were explored for men aged 20–49, comparing men who married during childhood and those who married in adulthood.

Self-reported data on the total number of children fathered, including those living in and outside the household as well as deceased children, were used to compare the size of men’s families both early and later in life based on whether they married in childhood or adulthood.^3^ The percentage of men aged 20–29 who biologically fathered three or more children at the time of the survey was compared according to whether they married by age 18 or in adulthood. The mean total number of children fathered and the mean number of living children were also compared for those married by 18 with those married in adulthood.

The linear relationship between men’s age and the number of children fathered was documented; additionally, the total number of children ever fathered by men who married during childhood and men who married in adulthood was compared according to the number of years men have been married. Since data on length of previous marriages were not collected, this analysis was restricted to currently married men aged 20–49 who reported to have been married only once to ensure accurate estimates of marriage duration.

For the 40–49 age group, additional analysis was conducted on whether men’s ideal family size varied according to age at marriage (before or at/after age 18).^4^ The percentage of men aged 40–49 who had already exceeded their ideal family size based on the number of living children at the time of the survey was compared between men based on their age at first marriage in bivariate and multivariate regression analyses.

### Statistical methods

Data were analysed using Stata, version 15.0. Analysis was weighted to account for the sampling design of each survey. Estimates based on fewer than 25 unweighted cases are not shown, and those based on fewer than 50 are noted. Results from the chi-square test for bivariate analysis and the Wald test for multivariate regression analysis along with confidence intervals (95%) were used to assess the strength of association and model fit. In the regression analysis used to explore the associations between age at marriage and each outcome-level variable, additional models were used to adjust for the effects of known confounders, specifically age as a continuous variable and household wealth as a categorical variable using quintiles.

## Results

### Sociodemographic background

Table 1 presents the countries with the highest percentage of men currently aged 20–24 who were married by age 18 based on the most recent available data. Prevalence of child marriage varies across five-year age groups, and trends appear inconsistent across countries. In 11 countries, prevalence appears higher among the youngest men (20–24) than the oldest (45–49), although the confidence intervals are wide and overlapping. In the remaining four countries, prevalence appears lower among the youngest cohort than the oldest, although this difference is only statistically significant in Nepal. The percentage of men currently aged 20–24 who were married by age 15 is considerably lower in all countries, suggesting that most child marriages involving boys occur during later adolescence, between the ages of 15 and 18 (Table 10 in Appendix).

**Table 1:** The percentage of men aged 20–49 who were married by age 18, by current age

Country	Year	Men’s current age						
		20–24	25–29	30–34	35–39	40–44	45–49	20–49
Bolivia (Plurinational State of) *N* = 3987 *P* = 0.550	2008	8.4 (6.1–11.4)	7.1 (5.0–10.0)	9.1 (6.8–12.0)	6.1 (4.3–8.5)	7.8 (5.4–11.1)	6.6 (4.5–9.7)	7.6 (6.6–8.7)
Central African Republic *N* = 3893 *P* = 0.010	2010	27.9 (24.2–32.0)	30.5 (26.2–35.2)	28.2 (24.2–32.5)	24.8 (20.5–29.8)	19.4 (15.8–23.6)	25.0 (20.3–30.4)	26.7 (24.8–28.7)
Comoros *N* = 1477 *P* = 0.396	2012	11.9 (8.0–17.2)	15.0 (10.5–20.9)	9.7 (6.1–15.1)	9.3 (5.6–14.9)	12.9 (8.0–20.2)	8.6 (4.9–14.8)	11.4 (9.2–13.9)
Cuba *N* = 3125 *P* = 0.848	2014	10.7 (6.9–16.1)	13.1 (8.8–19.3)	14.9 (9.8–22.1)	11.7 (7.4–18.1)	14.8 (10.5–20.5)	13.2 (9.0–18.9)	13.1 (11.1–15.5)
Guatemala *N* = 7242 *P* = 0.071	2015	9.6 (8.1–11.3)	11.8 (10.0–14.0)	11.8 (10.0–14.0)	11.6 (9.5–14.1)	14.3 (11.6–17.5)	11.5 (9.2–14.2)	11.5 (10.6–12.5)
Guyana *N* = 1308 *P* = 0.324	2014	8.5 (5.1–13.8)	6.1 (3.4–10.7)	5.2 (2.3–11.5)	9.8 (5.2–17.8)	4.0 (1.7–9.2)	4.9 (2.3–10.4)	6.6 (4.8–9.0)
Honduras *N* = 4815 *P* = 0.245	2011	12.2 (10.0–14.9)	14.5 (11.9–17.5)	13.4 (10.7–16.7)	16.7 (13.5–20.4)	11.6 (9.0–14.9)	13.1 (9.9–17.1)	13.6 (12.3–14.9)
Lao People’s Democratic Republic *N* = 7832 *P* = 0.013	2011	12.7 (10.8–14.9)	16.7 (14.6–19.0)	16.0 (13.7–18.7)	14.3 (12.4–16.5)	15.6 (13.1–18.5)	11.8 (9.9–14.2)	14.6 (13.5–15.7)
Madagascar *N* = 5293 *P* = 0.004	2013	12.9 (10.6–15.6)	12.3 (10.0–15.0)	8.6 (6.9–10.7)	9.7 (7.4–12.6)	7.1 (5.3–9.4)	10.6 (8.0–14.0)	10.4 (9.4–11.4)
Marshall Islands *N* = 704	2007	11.8	11.1	17.4	9.3	18.0	6.4	12.4
Mozambique *N* = 2627 *P* = 0.072	2011	8.7 (6.0–12.4)	10.7 (8.0–14.2)	7.3 (4.8–10.9)	5.6 (3.6–8.8)	3.7 (1.7–7.8)	6.0 (2.8–12.1)	7.6 (6.4–9.0)
Nauru *N* = 252	2007	12.3	17.7	7.5	27.4	7.1	6.5	13.8
Nepal *N* = 3131 *P* < 0.001	2016	10.3 (7.5–14.1)	13.3 (10.2–17.1)	19.9 (15.9–24.5)	22.7 (18.0–28.4)	21.3 (17.0–26.4)	18.6 (14.7–23.3)	17.3 (15.4–19.4)
Nicaragua *N* = 4650 *P* = 0.236	2011	16.2 (12.6–20.6)	16.7 (13.3–20.7)	18.7 (15.3–22.6)	20.9 (16.8–25.6)	21.9 (16.8–28.0)	14.9 (10.9–19.9)	18.0 (16.3–19.8)
Thailand *N* = 19783 *P* = 0.032	2015	10.1 (8.2–12.4)	7.5 (5.9–9.5)	5.7 (4.4–7.5)	8.2 (6.5–10.4)	8.9 (7.2–11.1)	7.8 (6.3–9.6)	8.0 (7.3–8.9)

Confidence intervals and *p* values are not shown for the Marshall Islands and Nauru because data sets were not publicly available

With the exception of the Central African Republic, prevalence of child marriage among men aged 20–49 is higher among those in the poorest household wealth quintile than among men in the richest (Table 2). Evidence of a strong association between household wealth quintile and marriage by age 18 is observed at the *p* < 0.01 level in eight countries (the Plurinational State of Bolivia, Guatemala, Honduras, Lao People’s Democratic Republic, Madagascar, Mozambique, Nepal, and Thailand).

**Table 2:** The percentage of men aged 20–49 who were married by age 18, by household wealth quintile, place of residence, and level of education attainment

Country	Household Wealth Quintile					Place of residence		Education	
	Poorest	Second	Middle	Fourth	Richest	Urban	Rural	None or primary	Secondary or higher
Bolivia (Plurinational State of)	8.3 (6.2–10.9)	9.8 (7.2–13.2)	9.9 (7.6–12.9)	6.6 (4.6–9.2)	4.2 (2.8–6.2)	7.6 (6.4–9.1)	7.4 (5.9–9.2)	11.5 (9.6–13.6)	5.4 (4.3–6.8)
*N* = 3987	*P* = 0.002					*P* = 0.842		*P* < 0.001	
Central African Republic	25.2 (22.0–28.6)	28.2 (25.2–31.5)	22.5 (19.3–26.0)	30.0 (24.9–35.7)	27.5 (22.1–33.7)	28.0 (24.1–32.3)	25.9 (24.0–27.9)	27.0 (25.0–29.2)	26.2 (22.9–29.8)
*N* = 3893	*P* = 0.140					*P* = 0.346		*P* = 0.670	
Comoros	17.1 (12.1–23.6)	10.3 (6.2–16.4)	10.0 (6.6–15.0)	11.9 (8.3–16.8)	9.0 (6.1–13.3)	10.2 (7.4–13.8)	12.0 (9.2–15.6)	13.6 (10.4–17.5)	9.3 (7.1–12.2)
*N* = 1477	*P* = 0.135					*P* = 0.417		*P* = 0.033	
Cuba	–	–	–	–	–	13.6 (11.3–16.3)	11.5 (7.7–16.9)	15.4 (6.7–31.5)	13.1 (11.0–15.4)
*N* = 3125						*P* = 0.459		*P* = 0.690	
Guatemala	17.1 (14.8–19.7)	14.2 (12.1–16.6)	13.2 (11.2–15.4)	10.2 (8.5–12.2)	5.5 (4.3–6.9)	8.9 (7.7–10.3)	13.7 (12.5–15.1)	16.7 (15.3–18.2)	5.2 (4.4–6.2)
*N* = 7242	*P* < 0.001					*P* < 0.001		*P* < 0.001	
Guyana	11.5 (7.6–17.2)	5.7 (3.2–9.7)	5.7 (3.0–10.6)	5.8 (2.9–11.0)	4.9 (1.8–12.5)	4.2 (2.2–8.1)	7.4 (5.2–10.5)	8.4 (4.0–16.8)	6.2 (4.4–8.6)
*N* = 1308	*P* = 0.176					*P* = 0.130		*P* = 0.459	
Honduras	15.7 (13.3–18.5)	13.4 (10.9–16.3)	16.3 (13.6–19.5)	12.3 (9.7–15.3)	10.2 (7.8–13.2)	13.1 (11.4–15.1)	14.0 (12.3–15.8)	16.9 (15.2–18.7)	7.9 (6.4–9.8)
*N* = 4815	*P* = 0.013					*P* = 0.493		*P* < 0.001	
Lao People’s Democratic Republic	22.8 (20.5–25.4)	20.8 (18.6–23.2)	15.9 (14.0–18.0)	9.5 (8.1–11.2)	6.5 (5.1–8.2)	7.3 (5.9–9.0)	17.5 (16.2–18.8)	20.9 (19.3–22.5)	7.7 (6.8–8.7)
*N* = 7832	*P* < 0.001					*P* < 0.001		*P* < 0.001	
Madagascar	15.0 (12.2–18.3)	13.2 (10.6–16.3)	9.7 (7.8–12.1)	10.5 (8.4–13.0)	6.6 (5.3–8.2)	6.0 (4.6–7.8)	11.4 (10.3–12.7)	13.8 (12.4–15.2)	4.4 (3.4–5.7)
*N* = 5305	*P* < 0.001					*P* < 0.001		*P* < 0.001	
Mozambique	6.5 (4.3–9.8)	11.1 (7.7–15.7)	11.7 (8.4–16.2)	6.6 (4.2–10.2)	3.5 (2.3–5.4)	6.7 (5.0–8.9)	8.1 (6.5–10.0)	9.0 (7.4–11.0)	3.7 (2.4–5.6)
*N* = 2627	*P* < 0.001					*P* = 0.304		*P* = 0.001	
Nepal	21.5 (17.3–26.5)	22.0 (18.5–25.9)	21.1 (17.6–25.0)	19.8 (15.3–25.2)	6.7 (4.7–9.7)	15.7 (13.2–18.6)	20.4 (17.7–23.4)	25.8 (22.5–29.3)	13.0 (11.0–15.3)
*N* = 3132	*P* < 0.001					*P* = 0.021		*P* < 0.001	
Nicaragua	–	–	–	–	–	16.9 (14.4–19.7)	19.5 (17.4–21.7)	24.7 (21.1–28.6)	15.8 (13.9–17.9)
*N* = 4650						*P* = 0.141		*P* < 0.001	
Thailand	7.3 (6.0–8.9)	10.2 (8.5–12.2)	9.6 (7.6–12.0)	8.8 (7.2–10.7)	4.0 (3.0–5.3)	8.1 (6.9–9.5)	8.0 (7.0–9.1)	11.0 (9.5–12.7)	6.4 (5.6–7.2)
*N* = 19783	*P* < 0.001					*P* = 0.900		*P* < 0.001	

Information on household wealth quintile unavailable for Cuba or Nicaragua

Prevalence is higher among men in rural locations compared to those in urban areas in all countries except the Plurinational State of Bolivia, the Central African Republic, Cuba, and Thailand; however, differences are statistically significant at the *p* < 0.01 level only in Guatemala, Lao People’s Democratic Republic, and Madagascar. In all countries with available data, the percentage of men aged 20–49 who married by age 18 appears higher among those with no education or only primary schooling compared to those with secondary education or higher; the differences in prevalence by level of men’s education are statistically significant at the *p* < 0.01 level in nine countries. Although prevalence among men with no education is higher compared to those who completed primary schooling, the confidence intervals are wide and overlapping (*not shown*).

### Sexual and reproductive health

#### Comprehensive knowledge of HIV

In all countries with data*,* the percentage of men aged 20–49 who lack comprehensive knowledge of HIV appears higher among men married as children than among those who married at/after age 18; these differences are significant at the *p* < 0.01 level in six countries (Table 3). Results from multivariate logistic regression analysis indicate that men who married as children in Lao People’s Democratic Republic, Madagascar, and Thailand have higher odds of lacking comprehensive knowledge of HIV compared to men who married at or after age 18 even after adjusting for their current age and household wealth quintile. This trend is also observed for the other countries with available data although not at the *p* < 0.01 level.

**Table 3:** The odds of lacking comprehensive knowledge of HIV, comparing men aged 20–49 who were married by age 18 with those married at or after 18 (reference group)

Country	Percentage of men who lack comprehensive knowledge of HIV		Unadjusted odds ratios (Model 1)		Odds ratios adjusted for age (Model 2)		Odds ratios adjusted for age and household wealth (Model 3)	
	Married by age 18	Married at/after 18	Odds ratio	*P* value	Odds ratio	*P* value	Odds ratio	*P* value
Bolivia (Plurinational State of) *N* = 2999	78.0 (71.2–83.6)	74.7 (72.3–77.0)	1.20 (0.82–1.76)	0.345	1.23 (0.84–1.80)	0.289	1.11 (0.74–1.66)	0.603
	*P* = 0.344							
Central African Republic *N* = 3455	76.1 (72.6–79.2)	75.0 (72.7–77.1)	1.06 (0.86–1.31)	0.585	1.05 (0.85–1.31)	0.633	1.10 (0.88–1.37)	0.405
	*P* = 0.585							
Comoros *N* = 1101	81.3 (73.7–87.1)	70.7 (65.9–75.0)	1.80 (1.12–2.91)	0.016	1.71 (1.06–2.74)	0.027	1.61 (1.01–2.58)	0.046
	*P* = 0.015							
Cuba *N* = 3125	41.6 (33.4–50.3)	36.6 (33.1–40.3)	1.18 (0.81–1.73)	0.391	1.18 (0.81–1.74)	0.387	–	–
	*P* = 0.273							
Guatemala *N* = 7242	81.2 (77.5–84.4)	72.4 (70.8–73.9)	1.53 (1.22–1.91)	< 0.001	1.52 (1.21–1.90)	< 0.001	1.18 (0.94–1.49)	0.159
	*P* < 0.001							
Guyana *N* = 1050	61.1 (44.2–75.7)	44.5 (40.0–49.3)	1.95 (0.95–4.02)	0.069	1.92 (0.93–3.96)	0.078	1.76 (0.91–3.45)	0.095
	*P* = 0.065							
Honduras *N* = 3788	68.6 (63.9–72.9)	66.7 (64.4–69.1)	1.08 (0.86–1.37)	0.492	1.12 (0.89–1.41)	0.345	1.03 (0.81–1.31)	0.831
	*P* = 0.492							
Lao People’s Democratic Republic *N* = 7832	78.3 (75.0–81.2)	68.0 (66.2–69.8)	1.69 (1.40–2.05)	< 0.001	1.69 (1.40–2.05)	< 0.001	1.32 (1.09–1.60)	0.005
	*P* < 0.001							
Madagascar *N* = 4236	85.1 (81.2–88.4)	73.2 (71.4–75.0)	2.10 (1.55–2.83)	< 0.001	1.97 (1.46–2.67)	< 0.001	1.87 (1.38–2.54)	< 0.001
	*P* < 0.001							
Mozambique *N* = 2627	55.6 (46.4–64.5)	48.3 (45.6–51.0)	1.28 (0.88–1.86)	0.202	1.34 (0.91–1.96)	0.136	1.29 (0.89–1.87)	0.184
	*P* = 0.121							
Nepal *N* = 2648	79.4 (75.1–83.1)	71.4 (68.5–74.2)	1.54 (1.14–2.08)	0.005	1.54 (1.14–2.09)	0.005	1.27 (0.94–1.71)	0.125
	*P* = 0.005							
Thailand *N* = 14583	57.4 (52.4–62.2)	48.7 (46.6–50.8)	1.42 (1.15–1.75)	0.001	1.40 (1.14–1.73)	0.002	1.33 (1.08–1.64)	0.008
	*P* = 0.001							

Nicaragua could not be included in this analysis because questions on knowledge of HIV included in that survey differed, making results incomparable with the standard indicator used

#### Knowledge and use of modern family planning methods

The percentage of men aged 20–49 with knowledge of at least one modern method of family planning is above 85% in all countries with available data, with little variation among men married as children and those who married as adults (*not shown*). Use of modern methods of contraception appears to vary widely across countries. In half of the countries with available data (the Plurinational State of Bolivia, Comoros, Honduras, and Nepal), the use of modern contraception is higher among men aged 20–49 who married by age 18 compared to those who married in adulthood, although differences are significant at the *p* < 0.01 level only in Comoros and Nepal (Table 4). With the exception of Madagascar, men aged 20–49 who were married by age 18 appear to have higher odds of using a modern method compared to men married in adulthood after adjusting for socioeconomic status and age in multivariate logistic regression analysis, although differences are significant at the *p* < 0.01 level only in Nepal.

**Table 4:** The odds of using a modern method of family planning, comparing men currently aged 20–49 who were married by age 18 with those married at or after 18 (reference group)

Country	Percentage of men using a modern method of family planning, by men’s age at marriage		Unadjusted odds ratios (Model 1)		Odds ratios adjusted for age (Model 2)		Odds ratios adjusted for age and household wealth (Model 3)	
	Married by age 18	Married at/ after age 18	Odds ratio	*P* value	Odds ratio	*P* value	Odds ratio	*P* value
Bolivia (Plurinational State of) *N* = 2999	34.9 (28.5–41.9)	32.6 (30.3–35.0)	1.11 (0.80–1.53)	0.535	1.01 (0.74–1.38)	0.959	1.07 (0.77–1.50)	0.674
	*P* = 0.535							
Comoros *N* = 1101	26.0 (18.8–34.8)	17.1 (14.2–20.6)	1.69 (1.11–2.59)	0.014	1.27 (0.82–1.95)	0.281	1.27 (0.82–1.99)	0.285
	*P* = 0.013							
Guatemala *N* = 5584	47.8 (43.6–52.0)	50.1 (48.3–51.9)	0.91 (0.77–1.09)	0.298	0.93 (0.78–1.11)	0.421	1.09 (0.91–1.30)	0.371
	*P* = 0.298							
Honduras *N* = 3789	70.2 (65.9–74.2)	65.6 (63.5–67.7)	1.23 (0.99–1.54)	0.058	1.25 (1.00–1.56)	0.049	1.29 (1.03–1.61)	0.027
	*P* = 0.058							
Madagascar *N* = 4235	24.3 (19.9–29.2)	32.2 (30.3–34.1)	0.67 (0.52–0.88)	0.004	0.65 (0.50–0.85)	0.002	0.69 (0.53–0.91)	0.008
	*P* = 0.004							
Mozambique *N* = 2223	18.3 (13.0–25.2)	18.6 (16.6–20.9)	0.98 (0.64–1.48)	0.906	0.94 (0.62–1.41)	0.752	1.17 (0.75–1.82)	0.486
	*P* = 0.906							
Nepal *N* = 2648	60.5 (55.3–65.5)	53.9 (50.9–57.0)	1.31 (1.05–1.63)	0.015	1.33 (1.07–1.66)	0.009	1.31 (1.06–1.63)	0.015
	*P* = 0.015							
Nicaragua *N* = 3609	73.9 (72.1–77.1)	74.7 (68.8–78.5)	0.96 (0.72–1.28)	0.790	0.96 (0.73–1.28)	0.790	–	–
	*P* = 0.790							

### Family size among the youngest and oldest cohorts of men

#### Early fatherhood

In nine of the ten countries with available data, the percentage of men aged 20–29 who had already fathered three or more children at the time of the survey is higher among men married as children compared to men married in adulthood (Table 5). In seven of the countries, more than a quarter of men aged 20–29 who married by age 18 reported having fathered three or more children. With the exception of Guyana, the odds of having fathered three or more children remain higher for men who married as children compared to men who married in adulthood even after adjusting for men’s current age and household wealth quintile, with differences observed at the *p* < 0.001 level. In all countries with data, both the mean total number of children fathered and the mean total number of living children appear higher among men aged 20–29 who married during childhood than those who married in adulthood; differences were observed at the *p* < 0.01 level in eight countries (Table 6).

**Table 5:** The odds of having fathered three or more children at the time of survey, comparing men aged 20–29 who were married by age 18 with those married at or after 18 (reference group)

Country	Percentage of men aged 20–29 who fathered three or more children, by men’s age at marriage		Unadjusted odds ratios (Model 1)		Odds ratios adjusted for age (Model 2)		Odds ratios adjusted for age and household wealth (Model 3)	
	Married by age 18	Married at/ after age 18	Odds ratio	*P* value	Odds ratio	*P* value	Odds ratio	*P* value
Bolivia (Plurinational State of) *N* = 780	31.4 (21.4–43.4)	14.5 (11.3–18.3)	2.69 (1.50–4.80)	< 0.001	5.37 (2.73–10.56)	< 0.001	6.17 (3.02–12.58)	< 0.001
	*P* < 0.001							
Comoros *N* = 279	30.3 (19.2–44.3)	10.2 (5.7–30.3)	3.84 (1.61–9.14)	0.003	5.63 (2.47–12.80)	< 0.001	7.25 (2.49–21.16)	< 0.001
	*P* = 0.002							
Guatemala *N* = 1807	31.9 (26.0–38.4)	12.4 (10.7–14.4)	3.30 (2.36–4.61)	< 0.001	6.80 (4.57–10.16)	< 0.001	6.88 (4.46–10.59)	< 0.001
	*P* < 0.001							
Guyana *N* = 316	6.3 (2.2–16.8)	6.3 (4.0–9.6)	1.00 (0.31–3.28)	0.997	1.26 (0.37–4.31)	0.710	1.28 (0.40–4.07)	0.675
	*P* = 0.997							
Honduras *N* = 1194	27.2 (21.8–33.3)	8.4 (6.7–10.4)	4.08 (2.82–5.90)	< 0.001	5.76 (3.82–8.68)	< 0.001	5.88 (3.84–9.01)	< 0.001
	*P* < 0.001							
Madagascar N = 1308	40.2 (33.0–47.7)	17.9 (15.1–21.0)	3.08 (2.13–4.45)	< 0.001	6.89 (4.41–10.76)	< 0.001	6.90 (4.27–11.1)	< 0.001
	*P* < 0.001							
Mozambique *N* = 811	60.0 (47.0–71.8)	21.8 (17.9–26.2)	5.39 (2.99–9.72)	< 0.001	11.10 (5.66–21.75)	< 0.001	10.14 (5.23–19.66)	< 0.001
	*P* < 0.001							
Nepal *N* = 718	36.3 (26.4–47.5)	7.2 (5.0–10.2)	7.37 (4.18–13.0)	< 0.001	13.61 (7.39–25.06)	< 0.001	11.33 (6.01–21.36)	< 0.001
	*P* < 0.001							
Nicaragua *N* = 1287	11.5 (7.8–16–8)	6.5 (4.7–8.8)	1.89 (1.09–3.25)	0.023	2.69 (1.53–4.75)	< 0.001	–	–
	*P* = 0.021							
Thailand *N* = 2381	7.5 (4.4–12.3)	1.6 (0.8–3.0)	5.03 (2.15–11.75)	< 0.001	8.75 (3.76–20.33)	< 0.001	9.39 (3.95–22.32)	< 0.001
	*P* < 0.001

**Table 6:** Among ever-married men aged 20–29 and 40–49, the mean number of children ever fathered and mean number of living children, by men’s age at marriage

Country	Ever-married men currently aged 20–29				Ever-married men currently aged 40–49			
	Mean number of children fathered		Mean number of living children		Mean number of children fathered		Mean number of living children	
	Married by age 18	Married at/ after age 18	Married by age 18	Married at/ after age 18	Married by age 18	Married at/ after age 18	Married by age 18	Married at/ after age 18
Bolivia (Plurinational State of)	2.09 (1.90–2.28)	1.48 (1.38–1.58)	1.97 (1.78–2.16)	1.39 (1.29–1.49)	6.42 (5.55–7.28)	4.52 (4.32–4.72)	5.38 (4.60–6.16)	3.98 (3.81–4.14)
	*P* < 0.001		*P* < 0.001		*P* < 0.001		*P* < 0.001	
Comoros	2.10 (1.03–3.17)	0.98 (0.73–1.23)	1.77 (0.90–2.64)	0.96 (0.71–1.20)	8.33 (6.25–10.42)	4.68 (4.11–5.25)	7.33 (5.96–8.70)	4.37 (3.87–4.87)
	*P* = 0.046		*P* = 0.077		*P* = 0.001		*P* < 0.001	
Guatemala	2.12 (1.96–2.28)	1.36 (1.30–1.41)	2.00 (1.85–2.16)	1.30 (1.25–1.36)	6.07 (5.68–6.46)	4.47 (4.31–4.64)	5.58 (5.22–5.95)	4.22 (4.06–4.37)
	*P* < 0.001		*P* < 0.001		*P* < 0.001		*P* < 0.001	
Guyana	0.90 (0.52–1.29)	0.78 (0.63–0.94)	0.87 (0.50–1.25)	0.75 (0.60–0.90)	3.88 (2.70–5.07)	3.31 (3.00–3.61)	3.66 (2.64–4.68)	3.14 (2.85–3.43)
	*P* = 0.556		*P* = 0.535		*P* = 0.358		*P* = 0.343	
Honduras	1.86 (1.70–2.02)	1.17 (1.10–1.24)	1.79 (1.63–1.95)	1.14 (1.07–1.21)	5.29 (4.72–5.87)	4.14 (3.96–4.32)	4.99 (4.47–5.51)	3.96 (3.79–4.12)
	*P* < 0.001		*P* < 0.001		*P* < 0.001		*P* < 0.001	
Madagascar	2.35 (2.14–2.56)	1.53 (1.44–1.61)	2.25 (2.05–2.44)	1.47 (1.39–1.56)	5.97 (5.28–6.66)	4.71 (4.54–4.88)	5.41 (4.79–6.02)	4.41 (4.25–4.56)
	*P* < 0.001		*P* < 0.001		*P* < 0.001		*P* = 0.002	
Mozambique	2.89 (2.56–3.21)	1.59 (1.47–1.72)	2.42 (2.12–2.72)	1.38 (1.27–1.49)	–	–	–	–
	*P* < 0.001		*P* < 0.001					
Nepal	2.19 (1.90–2.48)	1.02 (0.90–1.13)	2.00 (1.73–2.28)	0.97 (0.86–1.08)	4.45 (4.20–4.69)	3.43 (3.24–3.62)	3.99 (3.77–4.22)	3.07 (2.92–3.23)
	*P* < 0.001		*P* < 0.001		*P* < 0.001		*P* < 0.001	
Nicaragua	1.41 (1.26–1.55)	1.08 (0.98–1.17)	1.38 (1.24–1.52)	1.05 (0.96–1.15)	5.39 (4.80–5.98)	3.65 (3.39–3.91)	4.96 (4.42–5.51)	3.48 (3.23–3.73)
	*P* < 0.001		*P* < 0.001		*P* < 0.001		*P* < 0.001	
Thailand	1.24 (1.09–1.38)	0.79 (0.72–0.86)	1.23 (1.09–1.38)	0.78 (0.72–0.85)	2.17 (1.92–2.43)	1.78 (1.73–1.83)	2.12 (1.90–2.34)	1.75 (1.70–1.80)
	*P* < 0.001		*P* < 0.001		*P* = 0.002		*P* = 0.001	

For Mozambique, estimates for men aged 40–49 who were married by age 18 are based on fewer than 25 unweighted cases, so values are not shown

#### Fertility outcomes later in life

In eight of the ten countries with available data, the mean total number of children fathered and the mean number of living children fathered by men currently aged 40–49 were higher among men who married during childhood compared to those married in adulthood, with differences observed at the *p* < 0.01 level (Table 6). The largest difference was observed for Comoros, where men aged 40–49 who married by age 18 fathered 8.3 children on average compared to 4.7 for those married in adulthood (*p* = 0.001).

Linear regression analyses were conducted to explore the association between marriage by age 18 and the number of children fathered after adjusting for men’s current age and the number of years spent living in a marital or cohabiting union. Since data on length of previous marriages were not collected, analysis was restricted to currently married men who reported to have been married only once in order to ensure accurate estimates of marriage duration; the age group was expanded to include men aged 20–49 to allow for adequate sample size.

The total number of children fathered was first modelled as a linear regression for men aged 20–49, with marriage by age 18 (a dichotomous variable) and current age (a continuous variable) as potentially associated factors. The strong linear relationship between men’s age and the number of children fathered was documented (Table 11 in Appendix). A second model was then constructed to regress the total number of children on marriage by age 18 and marriage duration in years (a continuous variable), removing current age due to its collinearity with the number of years married (Table 7). The number of years a man remains married appeared positively associated with the number of children fathered in all countries at the *p* < 0.001 level. Once the number of years married remains fixed, the association appears inconsistent and weakened in eight of the ten countries with data.

**Table 7:** Results from linear regression analysis adjusting for the number of years married: Factors associated with the number of children fathered among currently married men aged 20–49

Country		Regression coefficient	*P* value	Confidence interval
Bolivia (Plurinational State of)	Marriage by age 18	−0.05	0.732	(−0.37–0.26)
	Years married	0.20	< 0.001	(0.19–0.22)
Comoros	Marriage by age 18	−1.05	0.030	(−2.01–-0.09)
	Years married	0.27	< 0.001	(0.24–0.31)
Guatemala	Marriage by age 18	0.35	< 0.001	(0.19–0.51)
	Years married	0.21	< 0.001	(0.20–0.21)
Guyana	Marriage by age 18	−0.34	0.345	(−1.06–0.37)
	Years married	0.12	< 0.001	(0.09–0.14)
Honduras	Marriage by age 18	−0.03	0.800	(−0.30–0.23)
	Years married	0.19	< 0.001	(0.17–0.20)
Madagascar	Marriage by age 18	−0.08	0.544	(−0.32–0.17)
	Years married	0.21	< 0.001	(0.20–0.22)
Mozambique	Marriage by age 18	−0.29	0.158	(−0.69–0.11)
	Years married	0.30	< 0.001	(0.28–0.32)
Nepal	Marriage by age 18	0.25	0.005	(0.08–0.44)
	Years married	0.13	< 0.001	(0.13–0.14)
Nicaragua	Marriage by age 18	0.11	0.414	(−0.15–0.36)
	Years married	0.16	< 0.001	(0.14–0.18)
Thailand	Marriage by age 18	−0.04	0.632	(−0.20–0.12)
	Years married	0.06	< 0.001	(0.06–0.07)

Results exclude men who report having been married or in union more than once

In the seven countries with available data, the mean number of children desired during one’s lifetime appeared consistently higher among men aged 40–49 who were married by age 18 compared to men married in adulthood (Table 8). These differences appear significant at the *p* < 0.01 level in the Plurinational State of Bolivia, Guatemala, Madagascar, and Nepal. In five countries, the average number of children fathered by men aged 40–49 appears higher than the average number of children desired for both men married as children and men married in adulthood (Tables 6 and 8).

**Table 8:** The mean number of children desired among ever-married men currently aged 40–49, by men’s age at marriage

Country	Married by age 18	Married at/after age 18
Bolivia (Plurinational State of) *N* = 996 *P* = 0.003	3.87 (3.36–4.38)	3.09 (3.00–3.22)
Comoros *N* = 316 *P* = 0.026	9.68 (6.88–12.47)	6.56 (5.88–7.26)
Guatemala *N* = 1616 *P* = 0.010	4.34 (4.01–4.67)	3.88 (3.74–4.03)
Honduras *N* = 1109 *P* = 0.541	4.19 (3.67–4.71)	4.02 (3.84–4.71)
Madagascar *N* = 1193 *P* = 0.001	7.33 (6.10–8.57)	5.29 (5.10–5.49)
Nepal *N* = 873 *P* < 0.018	2.61 (2.48–2.74)	2.43 (2.35–2.52)
Nicaragua *N* = 891 *P* = 0.343	4.27 (2.82–5.73)	3.56 (3.31–3.81)

For Mozambique, estimates for men aged 40–49 who were married by age 18 are based on fewer than 25 unweighted cases, so values are not shown

The percentage of men aged 40–49 who had already exceeded their ideal family size at the time of the survey appears higher among those married by age 18 compared to those married in adulthood in all countries with data; differences are significant in four of the seven countries. For men aged 40–49, the odds of having already exceeded one’s ideal family size appear higher among men who married by age 18 compared to those who married in adulthood even after adjusting for men’s current age and household wealth, with differences significant at the *p* < 0.01 level in Guatemala, Honduras, and Nepal (Table 9, Table 12 in Appendix).

**Table 9:** Odds of having exceeded one’s ideal family size based on the total number of living children at the time of survey, comparing men currently aged 40–49 who were married by age 18 with those married at or after 18 (reference group)

Country	Percentage of men exceeding their ideal family size		Unadjusted odds ratios (Model 1)		Odds ratios adjusted for age (Model 2)		Odds ratios adjusted for age and household wealth (Model 3)	
	Married by age 18	Married at/ after age 18	Odds ratio	*P* value	Odds ratio	*P* value	Odds ratio	*P* value
Bolivia (Plurinational State of *N* = 996	53.4 (39.2–67.1)	49.1 (45.1–53.1)	1.19 (0.65–2.16)	0.574	1.22 (0.68–2.20)	0.508	1.11 (0.60–2.06)	0.728
	*P* = 0.574							
Comoros *N* = 316	21.8 (10.8–39.0)	20.8 (14.5–28.9)	1.06 (0.42–2.67)	0.898	–	–	–	–
	*P* = 0.898							
Guatemala *N* = 1616	50.3 (42.9–57.8)	31.7 (28.7–34.8)	2.19 (1.57–3.05)	< 0.001	2.26 (1.62–3.15)	< 0.001	2.15 (1.54–3.01)	< 0.001
	*P* < 0.001							
Honduras *N* = 1109	48.3 (38.5–58.1)	33.5 (29.7–37.4)	1.85 (1.21–2.85)	0.005	1.87 (1.21–2.87)	0.005	1.81 (1.18–2.78)	0.007
	*P* = 0.004							
Madagascar *N* = 1193	22.4 (14.5–33.0)	21.3 (18.5–24.5)	1.07 (0.60–1.87)	0.821	1.05 (0.60–1.83)	0.872	0.97 (0.56–1.70)	0.921
	*P* = 0.820							
Nepal *N* = 873	69.3 (62.4–75.5)	44.7 (40.0–49.4)	2.80 (1.96–3.99)	< 0.001	3.02 (2.11–4.32)	< 0.001	2.63 (1.85–3.72)	< 0.001
	*P* < 0.001							
Nicaragua *N* = 848	53.2 (42.3–63.7)	30.9 (26.0–36.3)	2.54 (1.54–4.18)	< 0.001	2.62 (1.59–4.31)	< 0.001	–	–
	*P* < 0.001							

For Mozambique, estimates for men aged 40–49 who were married by age 18 were based on fewer than 25 unweighted cases, so values are not shown. For Comoros, estimates for men aged 40–49 who were married by age 18 were based on 35 unweighted cases, so additional models were not used

## Discussion

The 15 countries with the highest prevalence of child marriage among boys are geographically, economically, and culturally diverse, perhaps indicating country-specific drivers of the practice. Given the lack of a clear geographical pattern, further assessment of the extent of sub-national variation in prevalence is recommended to identify the most vulnerable populations. Moreover, for almost all countries included in this analysis, the legal age of marriage for men is 18 or older, with Bolivia and Thailand as exceptions; however, most countries permit marriage before age 18 based on specific circumstances, such as in cases of parental consent [27]. Future research on the enforcement of and exceptions to the legal age of marriage within these countries is warranted to better understand the social and cultural norms surrounding the practice. Although analysis of trends across the five-year age cohorts is limited by sample size, the absence of a consistent decline in prevalence of marriage by age 18 might suggest that the changes observed at the regional and global level do not reflect the situation in high-prevalence countries [6, 7].

Among the men who married during childhood, most entered into marital unions as older adolescents: across all countries, less than 3% of men currently aged 20–24 were married by age 15, with the exception of men in the Central African Republic (14%). For most countries included in this analysis, the patterns of prevalence observed by household wealth quintile, place of residence, and level of education attainment are aligned with findings for child marriage among girls [8]. Prevalence among men appears concentrated among those living in the poorest households and in rural areas, and among those with no education or only primary schooling. However, since data used for this analysis are from cross-sectional surveys, the results are unable to indicate whether these time-variant factors were antecedents or consequences of early marriage. Consequently, further research is needed on whether and to what extent child marriage negatively impacts boys’ educational and employment opportunities both in the immediate aftermath and later in life.

Similar to the findings on health outcomes of child marriage among girls, there is also evidence of reproductive health consequences for boys who marry during childhood. Even after adjusting for their current age and household wealth quintile, men who married during childhood appear more likely to lack comprehensive knowledge of HIV than their peers who married in adulthood. Girls who marry by age 18 are shown to be at higher risk of HIV infection due to their young age, physical immaturity, and limited power to negotiate safer sex [28, 29]. In the absence of data on HIV prevalence, these findings might suggest that men who marry during childhood are also at higher risk of infection.

There appears to be little variation regarding knowledge and use of modern methods of family planning among men aged 20–49 based on their age at first marriage. However, in most countries with available data, for both the youngest (aged 20–29) and oldest (aged 40–49) cohorts, the number of children fathered is consistently higher among men who married by age 18 than among men who married in adulthood. While this paper provides insight on the family size of young men, information on the exact timing and spacing of births could confirm whether men who married during childhood experienced an earlier onset of fatherhood compared to their peers. Given the high proportion of men aged 20–29 married by age 18 who reported having fathered three or more children, future studies might explore whether men who marry during childhood face greater responsibilities as parents and providers at an earlier age than men who delay marriage.

n four of the seven countries with available data (Honduras, Guatemala, Nicaragua, and Nepal), the odds of exceeding one’s ideal family size at the time of the survey appear higher among men aged 40–49 who married during childhood compared to their peers who married at older ages, even after adjusting for associated covariates. The strong linear relationship observed between marriage duration and the number of children fathered might indicate that the differences observed between men who married by age 18 and those who married in adulthood regarding both the number of children fathered and the percentage exceeding their ideal family size are related to longer exposure to being in a marital union.

Finally, future research on whether boys who marry as children are also more vulnerable to adverse mental health outcomes and risky behaviour than their peers would help guide programmes and policies aimed at meeting the needs of boys and young men who married during childhood. Beyond the sexual and reproductive health consequences noted for girls, evidence has revealed a negative association between child marriage and psychological well-being, suggesting girls married during childhood are at increased risk for depression and suicide [30–32].

### Limitations

The lack of nationally representative data on men’s age at marriage for all countries poses the greatest challenge in ensuring the list of countries with the highest prevalence is conclusive. Recall bias or mortality differences between men who marry in childhood and those who did so in adulthood might have affected the accuracy of prevalence estimates, particularly for the older age cohorts.

The types of household surveys drawn upon for this analysis are focused predominantly on females. Men’s questionnaires are not systematically included, thus restricting the sample size for this analysis. The 15–49 age group, which has been the focus of such surveys to accommodate women’s reproductive age span, has also been applied to men, thereby restricting analysis of men’s completed family size. The analysis of the number of children fathered remains inconclusive since men are not biologically restricted in their fertility and can continue fathering children beyond the oldest age covered in these surveys. Men who delay marriage might also delay fatherhood but still achieve the same family size as men who marry earlier, therefore distorting our comparison of the two groups’ total number of children.

## Conclusion

The overall lack of research on child marriage among boys has likely hindered the initiation and implementation of any large-scale programmatic and policy efforts to eradicate the practice. Results from this analysis help to bridge the evidence gap by providing a profile of younger and older men who married during childhood in the countries where the practice is most common. While this paper explores variation in men’s sexual and reproductive health and family size based on their age at first marriage, analysis of other short- and long-term outcomes for men who married by age 18 and their families can inform efforts to mitigate the effects for men who have already married in childhood. To protect the current and future generation of boys at risk of child marriage, further research is needed on the determinants of the practice, including whether the unions were arranged by third parties or initiated by the boys themselves, as well as any country-specific incentives.

## Data Availability

This current paper uses secondary data from the Multiple Indicator Cluster Surveys, Demographic and Health Surveys, National Institute of Statistics of Madagascar, and Encuesta Nicaragüense de Demografía y Salud. With the exception of the surveys for Madagascar and Nicaragua, all other data sets used for this analysis are currently housed in publicly available repositories: Demographic and Health Surveys (retrieved from <www.dhsprogram.com>) and Multiple Indicator Cluster Surveys (retrieved from <mics.unicef.org>). For Encuesta Nicaragüense de Demografía y Salud (2011–2012), access to the data set was granted to UNICEF through the National Institute of Development Information: <www.inide.gob.ni>. For Enquête Nationale sur le Suivi des Objectifs du Millénaire pour le Développement à Madagascar (2013), access to the data set was granted to UNICEF through the National Institute of Statistics of Madagascar: <www.instat.mg>.

## Appendix

**Table 10:** Percentage of men aged 20–49 who were married by age 15, by current age

Country	Men’s current age						
	20–24	25–29	30–34	35–39	40–44	45–49	20–49
Bolivia (Plurinational State of) *N* = 3987 *P* = 0.592	0.8 (0.3–2.1)	0.2 (0.0–0.6)	0.8 (0.3–2.5)	0.3 (0.0–1.3)	0.5 (0.1–2.2)	0.9 (0.3–3.2)	0.6 (0.3–1.0)
Central African Republic *N* = 3893 *P* = 0.062	14.0 (11.1–17.6)	11.9 (9.0–15.5)	11.4 (8.7–14.9)	10.9 (8.3–14.2)	6.9 (4.8–9.8)	9.6 (6.5–14.0)	11.2 (10.0–12.6)
Comoros *N* = 1477 *P* = 0.432	3.2 (1.5–6.5)	3.4 (1.5–7.5)	1.4 (0.4–5.3)	4.3 (2.1–8.7)	1.8 (0.6–5.6)	1.6 (0.5–4.7)	2.8 (1.8–4.1)
Cuba *N* = 3125 *P* = 0.167	1.1 (0.4–3.4)	3.1 (1.2–7.5)	2.4 (1.0–5.8)	2.6 (1.0–6.2)	1.5 (0.6–3.9)	0.4 (0.1–1.5)	1.7 (1.1–2.5)
Guatemala *N* = 7242 *P* = 0.156	0.8 (0.5–1.5)	0.8 (0.4–1.5)	1.0 (0.4–2.1)	1.8 (1.0–3.1)	0.9 (0.4–1.8)	0.5 (0.2–1.2)	1.0 (0.7–1.3)
Guyana *N* = 1308 *P* = 0.159	1.8 (0.5–5.6)	0.8 (0.3–2.1)	0.5 (0.1–1.9)	0.8 (0.2–3.2)	0.1 (0.0–0.9)	0.0	0.7 (0.4–1.5)
Honduras *N* = 4815 *P* = 0.597	1.7 (1.0–3.1)	2.4 (1.4–4.2)	2.8 (1.7–4.3)	2.1 (1.1–3.8)	1.4 (0.6–2.9)	2.9 (1.4–6.0)	2.2 (1.7–2.8)
Lao People’s Democratic Republic *N* = 7832 *P* = 0.397	2.6 (1.9–3.7)	3.0 (2.1–4.3)	4.3 (3.2–5.7)	3.2 (2.3–4.6)	3.8 (2.6–5.5)	3.0 (2.0–4.7)	3.3 (2.8–3.8)
Madagascar *N* = 5293 *P* = 0.600	1.8 (1.1–3.0)	2.3 (1.4–3.6)	1.9 (1.2–3.1)	1.9 (1.1–3.2)	1.0 (0.5–2.1)	2.1 (1.1–3.7)	1.8 (1.5–2.3)
Marshall Islands *N* = 704	0.0	0.0	0.0	0.0	0.0	0.0	0.0
Mozambique *N* = 2627	0.0	0.0	0.0	0.0	0.0	0.0	0.0
Nauru *N* = 252	0.0	0.0	0.0	0.0	0.0	0.0	0.0
Nepal *N* = 3131 *P* = 0.333	1.2 (0.5–2.8)	2.7 (1.5–4.8)	2.7 (1.3–5.5)	3.4 (2.1–5.5)	3.2 (1.9–5.4)	2.7 (1.5–4.7)	2.6 (2.0–3.3)
Nicaragua *N* = 4650 *P* = 0.214	2.0 (0.9–4.3)	1.8 (1.0–3.2)	2.4 (1.5–3.8)	3.3 (1.9–5.7)	3.8 (1.8–7.9)	0.7 (0.2–2.7)	2.3 (1.7–3.1)
Thailand *N* = 19783 *P* = 0.265	1.3 (0.8–2.1)	1.8 (1.0–3.4)	2.3 (1.5–3.4)	2.4 (1.6–3.7)	2.0 (1.3–3.0)	1.3 (0.8–2.1)	1.9 (1.5–2.3)

Confidence intervals and *p* values are not shown for the Marshall Islands and Nauru because data sets were not publicly available

**Table 11:** Results from linear regression analysis: Factors associated with the number of children ever fathered among currently married men aged 20–49

Model 1: Adjusted for men’s current age				
Country		Regression coefficient	*P* value	Confidence interval
Bolivia (Plurinational State of)	Marriage by age 18	1.37	< 0.001	(1.03–1.71)
	Current age	0.17	< 0.001	(0.16–0.18)
Comoros	Marriage by age 18	1.80	< 0.001	(0.88–2.72)
	Current age	0.17	< 0.001	(0.14–0.20)
Guatemala	Marriage by age 18	1.56	< 0.001	(1.39–1.72)
	Current age	0.17	< 0.001	(0.17–0.18)
Guyana	Marriage by age 18	0.78	0.035	(0.05–1.51)
	Current age	0.12	< 0.001	(0.10–0.15)
Honduras	Marriage by age 18	1.11	< 0.001	(0.82–1.39)
	Current age	0.16	< 0.001	(0.15–0.17)
Madagascar	Marriage by age 18	1.34	< 0.001	(1.06–1.58)
	Current age	0.17	< 0.001	(0.16–0.18)
Mozambique	Marriage by age 18	1.58	< 0.001	(1.22–1.94)
	Current age	0.26	< 0.001	(0.24–0.28)
Nepal	Marriage by age 18	1.16	< 0.001	(0.97–1.36)
	Current age	0.14	< 0.001	(0.13–0.15)
Nicaragua	Marriage by age 18	1.09	< 0.001	(0.83–1.35)
	Current age	0.14	< 0.001	(0.13–0.16)
Thailand	Marriage by age 18	0.51	< 0.001	(0.34–0.68)
	Current age	0.05	< 0.001	(0.05–0.06)

Results exclude men who report having been married or in union more than once

**Table 12:** Odds of having exceeded one’s ideal family size at the time of survey based on the total number of children ever fathered, comparing men currently aged 40–49 who were married by age 18 with those married at or after 18 (reference group)

Country	Percentage of men exceeding their ideal family size		Unadjusted odds ratios (Model 1)		Odds ratios adjusted for age (Model 2)		Odds ratios adjusted for age and socio-economic status (Model 3)	
	Married by age 18	Married at/after age 18	Odds ratio	*P* value	Odds ratio	*P* value	Odds ratio	*P* value
Bolivia (Plurinational State of) *N* = 996	67.3 (53.2–78.8)	56.3 (52.3–60.1)	1.59 (0.87–2.95)	0.132	1.64 (0.90–3.01)	0.172	1.54 (0.84–2.82)	0.164
	*P* = 0.129							
Comoros *N* = 316	28.4 (15.7–45.8)	23.7 (17.3–31.6)	1.27 (0.55–2.94)	0.569	1.33 (0.59–2.98)	0.484	1.04 (0.44–2.48)	0.916
	*P* = 0.568							
Guatemala *N* = 1616	57.2 (49.8–64.4)	37.4 (34.2–40.7)	2.23 (1.62–3.10)	< 0.001	2.31 (1.67–3.20)	< 0.001	2.13 (1.53–2.97)	< 0.001
	*P* < 0.001							
Honduras *N* = 1109	50.9 (41.0–60.7)	35.9 (32.1–39.9)	1.85 (1.20–2.85)	0.005	1.86 (1.21–2.87)	0.005	1.80 (1.17–2.77)	0.008
	*P* = 0.005							
Madagascar *N* = 1193	27.2 (18.7–37.8)	26.6 (23.5–30.0)	1.03 (0.62–1.72)	0.912	1.00 (0.60–1.67)	0.992	0.92 (0.55–1.54)	0.748
	*P* = 0.912							
Nepal *N* = 873	80.9 (74.5–86.0)	54.2 (49.2–59.1)	3.58 (2.36–5.41)	< 0.001	3.86 (2.54–5.87)	< 0.001	3.32 (2.21–5.0)	< 0.001
	*P* < 0.001							
Nicaragua *N* = 891	60.5 (49.7–70.4)	32.9 (27.9–38.3)	3.13 (1.90–5.15)	< 0.001	3.26 (1.98–5.36)	< 0.001	–	–
	*P* < 0.001							

For Mozambique, estimates for men aged 40–49 who were married by age 18 were based on fewer than 25 unweighted cases, so values not shown. For Comoros, estimates were based on 35 unweighted cases

## Abbreviations

DHS: Demographic and Health Surveys
MICS: Multiple Indicator Cluster Surveys
UNFPA: United Nations Population Fund
UNICEF: United Nations Children’s Fund
USAID: United States Agency for International Development

## Footnotes

1 Comprehensive knowledge of HIV was demonstrated by three items: correctly identifying the two major methods of preventing sexual transmission of HIV (frequent condom use and limiting sex to one faithful, uninfected partner); knowing that an individual who appears healthy can be HIV positive; and rejecting the two most common local misconceptions of HIV transmission. Questions used to assess men’s knowledge of HIV in the survey for Nicaragua differed from those used in other surveys; results are incomparable with the standard indicator used and not shown.

2 Use of a modern family planning method was defined as currently using at least one of the following: the pill, female and male sterilization, injectables, implants, male and female condoms, diaphragms, or emergency contraception. Knowledge of at least one modern family planning method was defined as having heard of at least one of the aforementioned methods. Information on men’s knowledge and use of family planning methods was not collected in the surveys for the Central African Republic, Cuba, Guyana, Lao People’s Democratic Republic, or Thailand.

3 Information on men’s total number of children fathered or total number of living children was not collected in the surveys for the Central African Republic, Cuba, or Lao People’s Democratic Republic.

4 To determine ideal family size, respondents who had living children were asked: “If you could go back to the time when you did not have any children and could choose exactly the number of children to have in your lifetime, how many would that be?” Information on men’s ideal family size was not collected in the surveys for the Central African Republic, Cuba, Lao People’s Democratic Republic, or Thailand.
